# Kinesio Taping as an Adjunct Therapy in Postoperative Care after Extraction of Impacted Third Lower Molars—A Randomized Pilot Study

**DOI:** 10.3390/jcm12072694

**Published:** 2023-04-04

**Authors:** Piotr Pławecki, Karolina Pierwocha, Wojciech Terlecki, Anna Kawulok, Mateusz Bogacz, Agnieszka Balicz, Magdalena Jędrusik-Pawłowska, Magdalena Dąbrowska-Galas, Tadeusz Morawiec

**Affiliations:** 1 Department of Dental Surgery, Faculty of Medical Sciences in Zabrze, Medical University of Silesia, Plac Akademicki 17, 41-902 Bytom, Poland; 2 Students Scientific Association, Department of Oral Surgery in Bytom, 41-902 Bytom, Poland; 3 Department of Kinesitherapy and Special Methods, School of Health Sciences in Katowice, Medical University of Silesia, 40-752 Katowice, Poland

**Keywords:** impacted tooth, kinesio taping, pain, edema, trismus, NSAIDs

## Abstract

Non-steroidal anti-inflammatory drugs (NSAIDs) are commonly administered according to protocol for the management of complications such as pain, swelling, and trismus following the removal of the third impacted lower molar; however, treatment with NSAIDs may result in multiple adverse effects. The aim of this study was to compare the effectiveness of kinesio taping (KT) and the use of NSAIDs in the treatment of postoperative complications after extraction of an impacted third lower molar. Material and methods: The study comprised a group of 30 patients, randomly divided into the test group (with KT, n = 15) or the control group (without KT, n = 15). The surgery was performed according to standard procedures. In the test group, KT was applied immediately after surgery. Pain, swelling, and trismus were assessed. The VAS scale was used to assess pain. Swelling was measured based on six reference points on the face using a tailor’s meter, and a caliper was used to measure the distance between the upper and lower medial incisors of the upper and lower teeth to determine the extent of trismus. Measurements were performed three times: on the day of the surgery, on the second day following the surgery, and on the 7th day after the surgery. Results: Pain intensity (day of procedures), maximum mouth opening (on the seventh day after the surgery), and the use of NSAIDs (day of surgery) were significantly lower (*p* < 0.05) in the test group than in the control group. Conclusions: Kinesio taping in addition to NSAIDs was found to be more effective than NSAIDs alone in increasing the degree of jaw opening, decreasing pain intensity, and reducing the non-steroid anti-inflammatory dosage in patients after impacted mandibular wisdom teeth surgery.

## 1. Introduction

Surgical removal of the impacted mandibular third molars is a dental procedure performed by dental surgeons. However, it bears a high risk of postoperative complications such as pain, swelling, and trismus; thus, all contraindications and possible postoperative complications should be analyzed [1]. Moreover, aspects of surgical procedures, such as incision of the mucosa, retraction of the mucoperiosteal flap by the surgical hook, or detachment of the flap, can exacerbate trauma experienced by patients [1]. It can be highly burdensome, with the procedure itself potentially impairing the patient’s daily routines [1]. Administration of non-steroidal anti-inflammatory drugs, such as ibuprofen, ketoprofen, or naproxen, is common practice to alleviate complications and has proven effective, yet the drugs show multiple side effects [2,3]. The most common complications affect the digestive system, affecting up to 30% of patients receiving non-steroidal anti-inflammatory drugs (NSAIDs). Moreover, upper gastrointestinal bleeding has been reported as a consequence of the first dose of NSAIDs. In the USA, gastrointestinal tract complications related to NSAIDs are the cause of more than 20,000 deaths annually (similar records were observed for asthma, neck tumors, and melanoma) [3]. Other complications include disorders of the excretory, respiratory, and cardiovascular systems. From this perspective, in addition to conventional therapies, new alternatives are being sought, with an increasing focus on the use of both natural and synthetic products. *Cannabis sativa* L. represents an interesting source of bioactive compounds, including non-psychoactive cannabinoids, flavonoids, and terpenes, many of which are effective in reducing pain intensity [4]. Persistent orofacial pain, especially that caused by temporomandibular disorders, may be treated with the use of botulinum neurotoxin. Botulinum toxin has emerged as a popular option for patients with myofascial TMD who do not completely recover from their condition after receiving conservative care and medication [5].

Another example of an alternative method of orofacial pain relief is the use of kinesio taping. The application of tapes does not limit the range of mobility and therefore does not impact daily routines. The tape works upon the skin-fascia correlation, facilitating mutual displacement of the tissues, while facilitating subcutaneous flow and eliminating stagnation as well as lymphatic edema [6]. 

Kinesio taping (KT) is a non-invasive method developed in Japan to support both treatment and rehabilitation [6,7]. It has become a popular method applied to support the management of multiple types of injuries; however, in the region of Silesia where the research has been conducted there is little awareness of this method. The wide range of applications was comprehensive, as described by Kase K., Kase T., and Wallis [6]. The procedure is used both in post-traumatic care and prevention due to it being non-invasive, well-tolerated by the patients, and having proven to be effective in alleviating complications related to the inflammatory response [3,8,9].

Tapes gently lift the skin and decompress underlying soft tissues through the formation of convolutions; thus, the circulation in subcutaneous space is improved. Moreover, stimulation of skin mechanoreceptors blocks the signal due to gate control theory, resulting in pain alleviation [10,11]. Kinesio taping has become the focus of interest in multiple dental areas, including the treatment of trigger points, temporomandibular joint dysfunction, and dental surgery [12]. In particular, the procedure is frequently applied as the postoperative protocol in the surgical removal of the impacted third molar, alleviating troublesome complications such as trismus, pain, and swelling [13,14]. The available literature documents outline the effectiveness of taping in swelling reduction, postoperative care of patients with stabilized zygomatic-orbital fractures [15], and after orthognathic surgeries [16,17]. Therefore, it is possible that the application of kinesio taping could be an alternative method of managing the inflammatory process in order to reduce NSAID intake. In addition, the implementation of kinesio taping in the treatment of disorders and pain management in patients with temporomandibular joint dysfunction [18,19] and sleep bruxism [20] has now become more and more popular. In the literature, there have been three meta-analyses proving the effectiveness of kinesio taping in dental surgery [21,22,23]. However, their results are ambiguous. Therefore, the aim of our study was to compare the effectiveness of kinesio taping (KT) in addition to NSAIDs with the use of NSAIDs alone in the treatment of postoperative complications after extraction of the impacted third lower molar. 

## 2. Materials and Methods

A randomized study was conducted to compare the effect of the application of kinesio taping vs. no taping on patients after the extraction of the lower third molar.

Power analysis

A sample size was estimated for the main outcome measure, the change in MMO (maximal mouth opening), considering the results of the study by Tatli et al. [24] (the required minimum standardized mean difference was set to 1.002). To obtain a power of research of 0.80 and the desired statistical power of 0.05, 20 patients were required, which resulted in a minimum of 10 patients per group. Considering potential data loss due to patient dropout, the sample size was increased by 50%, resulting in 15 patients in each group, for a total of 30 patients.

Randomization

30 patients, aged between 16 and 64, that met the inclusion criteria were randomly assigned to one of the two study groups using a random number generator (APA: Haahr, M., 19 March 2023). RANDOM.ORG: True Random Number Service [25]).

**Figure 1:**
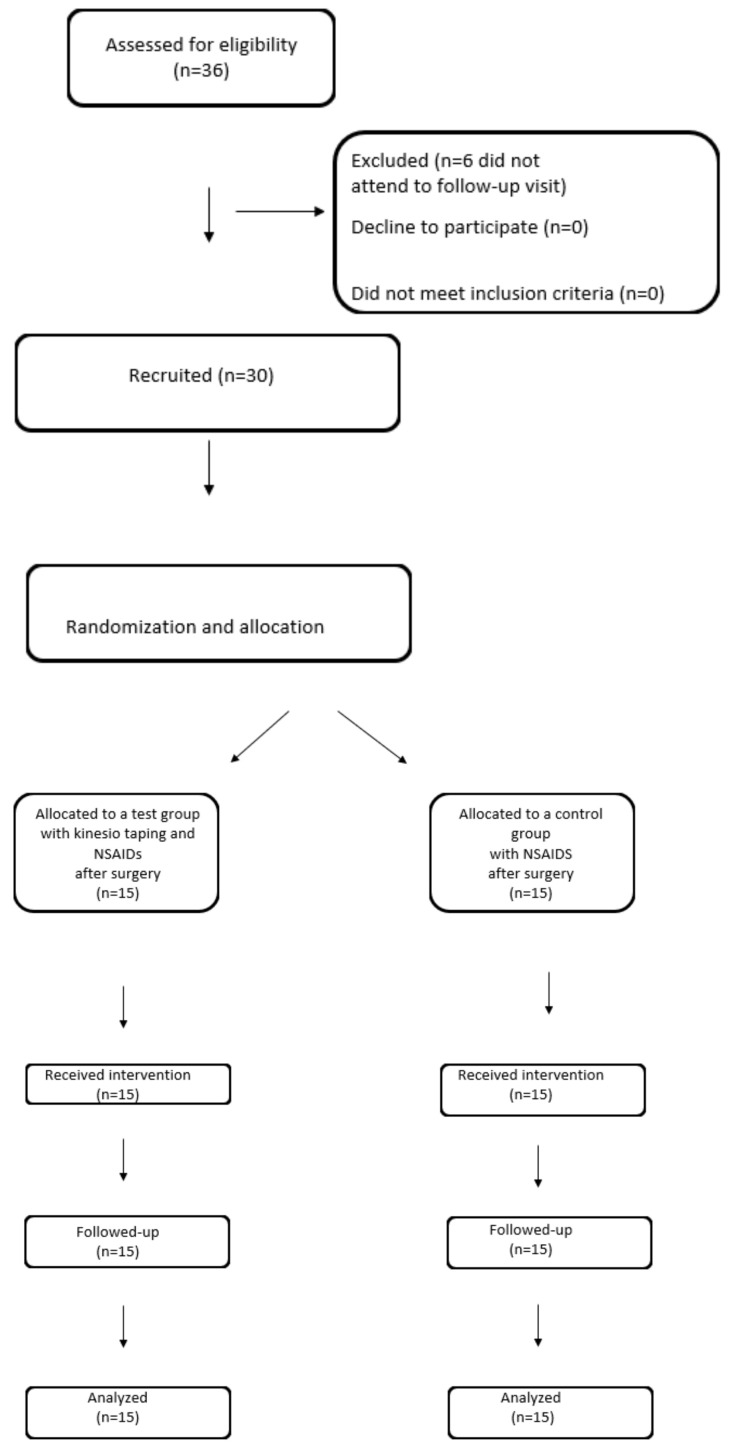
Flow chart of the study design.

Even-numbered patients were assigned to the test group (with kinesio taping application, n = 15), and those with an odd number were assigned to the control group (without kinesio taping application, n = 15) (Figure 1).

The research took place between November 2020 and June 2021 in the Department of Oral Surgery at the Medical University of Silesia in Katowice, Bytom, Poland.

The study was approved by the Bioethical Committee of the Medical University of Silesia in Katowice (Resolution no. PCN/022/KB1/56/20).

### 2.1. Participants

Consecutive patients were selected based on the following criteria [26]: patients without comorbidities were qualified for surgical extraction of the impacted lower wisdom teeth; patients with indications for surgery to remove the impacted lower wisdom tooth for orthodontic reasons; patients with indications for surgery to remove the impacted lower wisdom tooth for surgical reasons (inflammation/pathological changes); and patients with indications for surgery to remove the impacted lower wisdom tooth for prophylactic reasons. The exclusion criteria concerned: patients with a chronic history of comorbidities; patients undergoing permanent pharmacological treatment; use of stimulants; pregnancy; and patients undergoing treatment and diagnosed with head and neck tumors. To comply with ethical standards, informed consent was obtained from all the participants enrolled in the study. Inferior third molars have been classified according to the Ganss ratio [27] to determine the complexity of the surgical extraction (Table 1).

### 2.2. Procedures

In each participant the lower third molar was extracted and the level of swelling, degree of jaw opening, and pain were measured. In the test group, kinesio taping was applied (Figure 1).

The surgical procedures to extract the lower third molar were carried out in patients of both groups by the same operator, using a repeatable technique.

**Figure 2:**
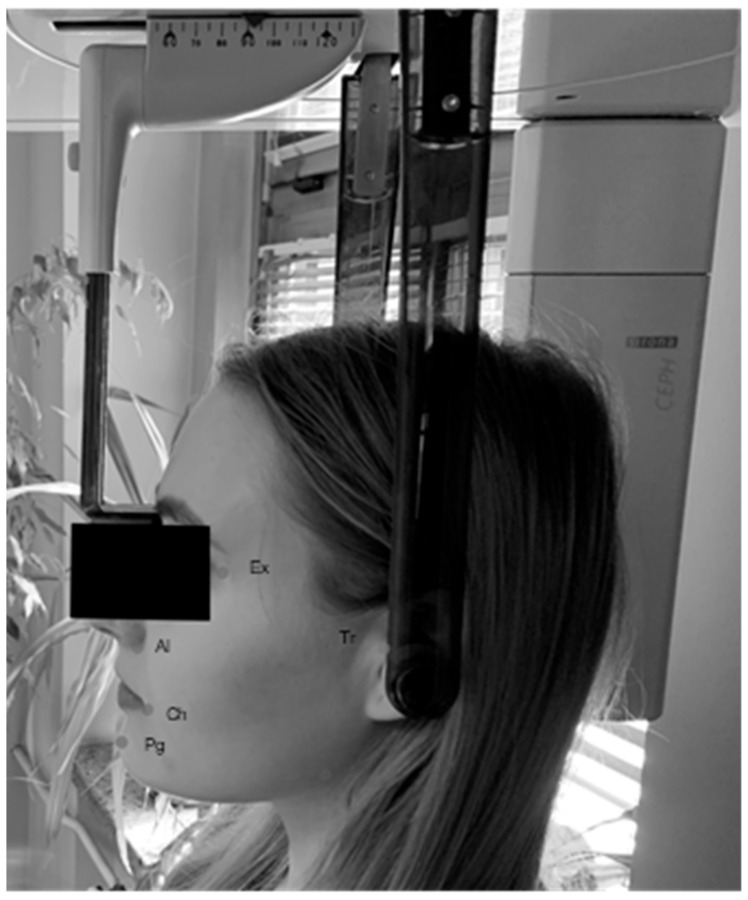
Stabilization of the patient’s head for repeated measurements.

Infiltration and conduction anesthesia were administered to the mandibular foramen with two ampoules of Lignocainum hydrochloricum 2% cum noradrenalin 0.00125% (by WZF Polfa S.A., Warsaw, Poland), then the mucoperiosteal flap was dissected from an angular incision. A small amount of bone was removed from the outside with a drill. The exposed tooth was either removed completely after separation of the crown from the roots. The bone bed was cured, and in case of any pathological changes, the specimen was sent for histopathological examination. The post-extraction wound was sutured using non-absorbable nylon surgical sutures—Braun Dafilon—USP 4/0, DS. 24.75 cm, 3/8 circle (B. Braun Melsungen AG., Melsungen, Germany), and a pressure dressing was applied for approximately 30 min. Each surgical procedure to remove the third lower molar was preceded by measurements taken in the clinical room. A cephalostat was used to obtain the repeatable position of the head, adjusted and recorded individually for each patient, followed by metric measurement of the facial integuments in relation to specific reference points and measurement of the degree of mouth opening. The patient’s head was stabilized for repeated measurements (Figure 2).

#### 2.2.1. Numerical Determination of the Degree of Swelling

A tailor’s meter was used to determine the degree of swelling based on the measurable parameters (Figure 1). Five distances (in mm) were measured on the basis of six reference points on the face from the angle of the mandible, including: (1) the tragion skin point (Tr), (2) the outer corner of the eye (Ex-exocanthion), (3) the wing of the nose (Al-alare), (4) the lip corner (Ch-cheilon), and (5) the pogonion skin point (Pg). The degree of edema was determined for each patient as the sum of the measured distances. Measurements were made before the procedure and during follow-up at two and seven days after the procedure.

#### 2.2.2. Jaw Opening Degree

In order to determine the occurrence of trismus, a caliper was used to measure the distance (mm) between the upper and lower medial incisors of the upper and lower teeth. Measurements were taken before the procedure and during follow-up at two and seven days after the procedure.

#### 2.2.3. Assessment of Pain on the VAS Scale

The VAS scale was used to record the subjective pain sensations of patients in both groups. The VAS scale is a visual analogue scale that measures pain intensity. It consists of a 10 cm line with two end points that represent 0 (‘no pain’) and 10 (‘pain as bad as it could possibly be’) [28]. The results can be used to monitor changes in pain intensity over time and to compare pain intensity between patients with similar medical conditions. While there are conflicting views on the advantages of the VAS over other pain imaging techniques [29], it is still widely used [30]. The patients were asked to rate their current level of pain by placing a mark on the line. Measurements were taken on days two and seven after the procedure, considering the degree of pain experienced by the patient on the day of the procedure. In the event of excessive pain, the patient was required to evaluate the perceived pain level on the VAS scale before taking any analgesic.

#### 2.2.4. Application of the Kinesio Tape

**Figure 3:**
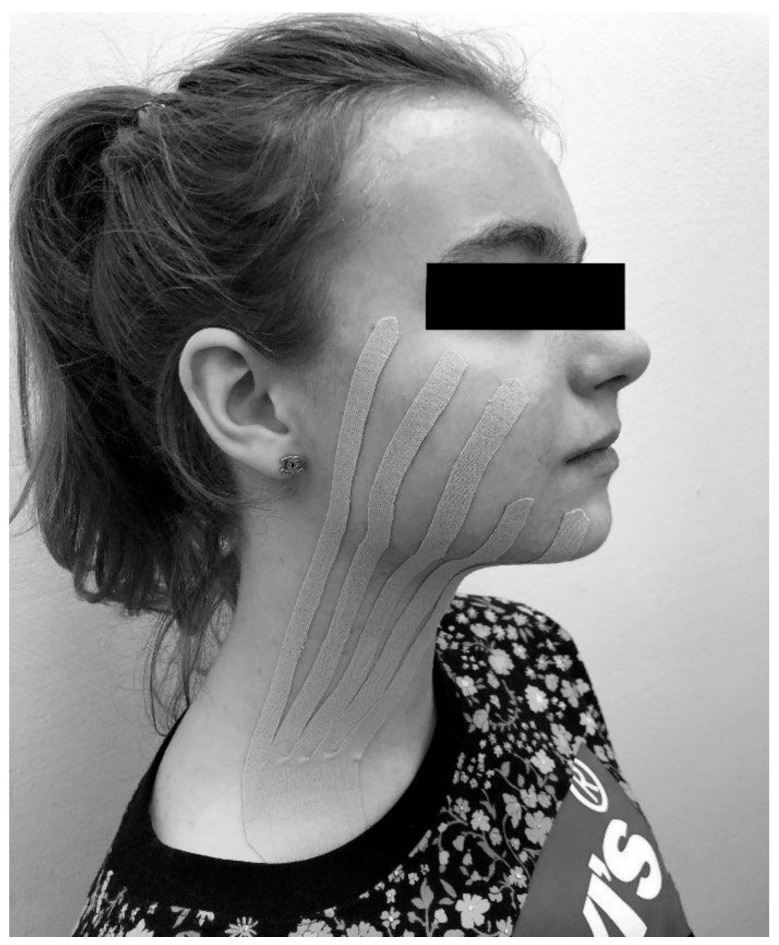
An example of the taping method performed on a test group patient.

All of the procedures carried out within the study to evaluate the effectiveness of kinesio taping after extraction of the third lower molar were carried out by the same physiotherapist with the use of Kinesio Tex Classic tape (dimensions 5 cm × 4 m, beige). The length of the tape was measured individually for each patient, starting from the subclavian fossa and ending at the zygomatic arch. The tape was cut into five equal strips of 1 cm in width each to form the shape of a fan. Before sticking the tape, the patient’s skin was cleaned, degreased, and dried (if necessary, also shaved at the point of gluing). The KT tape was placed to form a single fan over the area of expected edema (Figure 3). The skin surfaces on the side of the procedure were covered, starting from the supraclavicular fossa, through the mandible, and ending with the swollen buccal area. The base of the tape was placed over the subclavian lymph nodes in the direction of lymphatic drainage. The cropped strips were directed according to the branching of the lymphatic vessels, reaching the zygomatic arch. With the tape positioned in place, each patient was instructed to carry out daily activities during the following 5 days with no excessive care of the dressing. The patients were also recommended to maintain the tape for a minimum of 3 days and up to 5 days. If required to remove the tape earlier due to an allergic skin reaction, the patients had been instructed on how to remove the dressing safely with the use of oil.

#### 2.2.5. Post-Surgical NSAID’s Administration

Both Groups were instructed to take 100 mg of Ketoprofenum—Ketonal Forte in case of pain as needed.

### 2.3. Statistical Analysis

Statistical analyses were performed using the Python 3.7 (Python Software Foundation. Python Language Reference, version 3.7) and SciPy 1.4.1 (SciPy 1.0: Fundamental Algorithms for Scientific Computing in Python. Descriptive analyses, Shapiro-Wilk tests, and chi-squared tests were used to compare the participant’s baseline characteristics. One-way ANOVA for data transformed using box-cox (where appropriate) and Kruskal–Wallis one-way ANOVA tests were used to evaluate differences among the groups at the same time interval. The level of significance was *p* < 0.05.

## 3. Results

The mean age of participants in the test group was 22.93, and in the control group it was 29.73. There were nine women and six men in the test group, and twelve women and three men in the control group (Table 2).

No allergies were encountered. Each patient followed the recommendations for the period of the KT application. In addition, no patients removed the KT earlier than recommended. When comparing the maximal mouth opening at baseline, there was no significant difference between the two groups (*p* = 0.128). On the second and seventh days after surgery, the ratio of maximal mouth opening compared to the original maximal mouth opening was calculated, and we found that on the second day, the difference between the groups was not statistically significant (*p* = 0.073). On day 7, the difference between the groups was statistically significant (*p* = 0.0101). KT values were higher than those in the control group during both follow-up visits (Table 3).

On the first day after the surgery, the sensations measured with the subjective VAS scale were significantly lower in the KT group (*p* = 0.0114), with a significant decrease in pain in both groups when compared to their respective baseline measures. On days 2 and 7, the differences between the groups were not statistically significant (*p* = 0.391 and *p* = 0.405, respectively) (Table 4).

Facial swelling measurements were compared as the sum of all drawn lines. Baseline face measurements in the groups were compared (*p* = 0.765). On the second and seventh postoperative days, the ratio of the sum of all the lines drawn to the sum of the baseline face measurements was calculated, which revealed no significant differences between the groups (*p* = 0.227 and *p* = 0.963, respectively) (Table 5).

On the first and second day after the surgery, the amount of medication taken was significantly lower in the KT group (*p* = 0.0102 and *p* = 0.0131, respectively). On the seventh postoperative day there was no statically significant difference (Table 6).

## 4. Discussion

The aim of our study was to compare the effectiveness of kinesio taping (KT) in combination with NSAIDs with the use of NSAIDs alone in the treatment of postoperative complications after extraction of the impacted third lower molar. Analysis of the results showed a significant difference in mouth opening seven days after surgery (*p* = 0.0101).

The use of a caliper and the precision assumed by the measuring technique ensured the highest reproducibility and reliability of the results. This measurement proved to be the most important in our study. In the test group, the percentage change of this parameter was significantly smaller than among the controls, proving the effectiveness of the tested method.

Post-extraction pain reduces the patients’ quality of life, and it is an indication for taking analgesics, especially NSAIDs. There is a great need to introduce pain-reducing treatment, especially using methods without side effects. Kinesio taping is one such treatment. The results of our study showed that the average pain complaints assessed by the patients on the VAS scale were significantly lower (*p* = 0.0114) in the test group one day after surgery. The measurement of pain assessed by the patients on the VAS scale and the amount of analgesics taken were statistically significant in our study. The VAS scale is an internationally recognized tool that allows the patient to easily assess their pain. The results of our study showed that the average pain complaints assessed by the patients on the VAS scale were significantly lower in the test group (*p* = 0.0114) compared to the control group, one day after surgery. Moreover, patients from the test group reported a significantly lower intake of NSAIDs one day after surgery (*p* = 0.0102) and two days after surgery (*p* = 0.0131) when compared to a control group.

In our study, no significant difference was observed between the control and test groups with regard to facial tissue swelling.

The measurement of edema was not markedly higher in the study group than in the control group. Kasprzyk-Kucewicz et al. proved in their study that thermal imaging can be helpful in the assessment of the postoperative condition in dental surgery, and its usage may be considered in studies examining the effectiveness of kinesio taping [21].

In 2019, Tristão da Rocha Heras et al. observed a small group of patients showing a positive effect of KT on reducing the side effects of surgical extraction of the impacted wisdom teeth [31]. This encouraged us to increase the number of patients and re-examine their thesis. Contrary to Tristão da Rocha Heras et al., our study compared the measurements carried out after a single operation to remove the impacted molar in the test group of 15 patients subjected to taping and among 15 controls following the standard postoperative care protocol.

In the study above, patients who underwent surgical removal of the third molar were evaluated during the postoperative period. Four parameters were assessed: trismus, measured by the maximum opening of the mouth; swelling; pain intensity on the VAS scale; and the amount of analgesics taken by the patients throughout the postoperative period. The parameters were assessed equally in both the control and the test groups.

In their study, Ristow et al. [32] did not use any standard analgesics, such as paracetamol or ibuprofen, during the postoperative period in either the test patients or the controls who underwent surgical removal of the third molar. Both groups received a single postoperative dose of 2000 mg ampicillin or 1000 mg sulbactam, and postoperatively all patients received 50 mg diclofenac every 8 h for 3 days. In this study, the subjective satisfaction index of patients after the surgery was significantly higher in the group treated with kinesio taping.

In his studies on the use of KT in patients after orthognathic surgery and repositioning of orbital-zygomatic fractures [15,33], the same author prescribed intensive analgesic treatment, including NSAIDs, for patients in both the control and test groups (1000 mg of paracetamol twice a day and 600 mg of ibuprofen once to three times a day). The probable reason was greater intensity of pain expected given the profiles of patients qualified for these procedures. Nevertheless, Ristow et al. reported that the postoperative comfort of the test group patients, receiving NSAIDs along with kinesio taping, was significantly higher than that of the controls.

Analyzing the subsequent study by Tristão da Rocha Heras et al. evaluating the use of KT in patients after surgical extraction of the third molar [31], attention should be paid to the use of a complete analgesic therapy protocol in both the control and the test groups. During the postoperative period, each of the patients received 750 mg of paracetamol every 8 h, 4 mg of dexamethasone every 12 h during the first two days (the first dose one hour before the procedure), and 500 mg of ampicillin every 12 h for seven days (the first dose one hour before the procedure). Analysis of the results showed a significantly higher reduction in pain intensity perceived by the test group patients who additionally received kinesio taping, as compared to pain intensity among the controls. Although the role of KT therapy in pain suppression during the postoperative period has been confirmed, the authors emphasize the need for further research in order to verify this process.

Taking into consideration the respondents’ comfort, our study made it possible to take NSAIDs in the event of pain, while the patients were requested to assess their pain with the use of the VAS scale before medication. This ensured the assessment of this parameter and, at the same time, the observation of the amount and changes in the demand for analgesics in each of the patients. The authors of the recent study did not decide to administer dexamethasone, as performed by Tristão da Rocha Heras and colleagues.

Jaroń et al. in their study from 2021 measured three parameters: trismus, swelling, and pain in two groups who underwent surgical removal of an impacted third lower molar. Both groups used the same pharmacological postoperative protocol. The patients were instructed to use ketoprofen in a 100 mg dose taken twice daily. The patients in the study group had the kinesio tape applied immediately after the surgery. The 50 mm-wide tape was cut into three separate pieces of equal width. The study showed a significant decrease in trismus and pain sensation on the third and seventh days after surgery and a significant decrease in facial swelling on the third day after surgery [34].

Al-Shamiri et al. studied the effectiveness of dexamethasone, emphasizing the beneficial effect of preoperative or postoperative oral administration of dexamethasone in the management of postoperative complications following lower third molar surgeries, including pain, swelling, and trismus. It may be concluded that oral application of dexamethasone before the surgery substantially reduces pain sensation [35].

Yurttutan and Sancak [36] also analyzed pain and edema after surgical extractions of lower third molars using the visual analogue scale, which is considered one of the best measurement methods. The study showed significantly lower pain scores and reduced use of analgesics during the postoperative period in the KT group. The results are in line with our findings and those quoted earlier.

In their study, Gözlüklü, Ulu, and Yilmaz [37] compared the taping techniques—split-mouth and single-blinded—applied to two groups of patients. The controls, in whom, no kinesio taping was used also participated. VAS was used to score pain, while the 3dMD face system was applied to measure swelling.

Interestingly, the results showed that the new taping technique provided more effective pain relief than the standard one. The swelling assessment using the face system can also be more effective than the classic method used in previous studies.

Of note is the latest study by Tatli et al. [24] investigating the effectiveness of kinesio taping when compared to the placebo and control groups after third molar surgery. In the KT group, swelling, pain, and trismus were significantly reduced on the 2nd and 4th postoperative days as compared to other groups. Nevertheless, the authors emphasize that the outcomes for the placebo and control groups were comparable. The results obtained showed that kinesio taping was an effective method to reduce morbidity after impacted mandibular third molar surgery. Observations from studies also reveal that placebo taping is definitely less effective than taping in the correct manner, thereby justifying the application of the proper taping technique.

There is no consensus among the researchers regarding the duration of kinesio taping. Genc and Erdil kept tapes for two days [38,39]; Jaroń [34], Tatli [24], Gözlüklü [37], de Rocha Heras [31], and Ristow [33] kept tapes for five days; and Yurttutan [36] kept tapes for seven days after the surgery.

At present, three meta-analyses have been published, however the results are ambiguous. A paper by Firozi et al. [21] where he included nine randomized clinical trials with 444 participants showed a statistically significant reduction in pain and swelling scores before the seventh postoperative day. On the seventh postoperative day, no significant difference was observed between the KT and control groups in terms of pain and swelling. Additionally, KT led to an increase in patients’ maximum mouth opening by more than 3 mm in postoperative intervals. KT was effective in reducing postoperative pain within the first 48 h after surgery and improving mouth opening during all postoperative intervals.

Wang, Zhu, Guo, and Sun [23] conducted a meta-analysis comprising eight articles. The results revealed that, compared with the control group, the application of KT reduced pain in the early and late postoperative periods and therefore reduced the dosage of analgesics taken by patients.

Qi et al. [22] compared eight studies comprising 453 participants in total. This meta-analysis seems to be the closest to our results and showed a significant reduction of postoperative pain, but no significant difference was observed in terms of swelling between both groups. Another method used in the management of postoperative complications following the lower third molar surgeries is the application of a Hilotherm face mask to maintain a low temperature. Studies by Frank [40] showed a statistical reduction in the size and duration of edema in patients subjected to 2-h of cooling of the operation site at 12 °C, when compared to the controls. The pain sensation in patients undergoing local hypothermia was also significantly lower than in those undergoing the same procedure without cooling. Despite being effective, the method is costly and requires specialized infrastructure.

After offering postoperative kinesio taping care to a wide group of patients, only a small portion of them agreed to participate in our study, which we believe is due to the low level of knowledge about kinesio taping in our region. With our research, we want to popularize kiensio taping among patients in the region of Silesia, which in the future will allow us to gather a larger research group.

In summary, kinesio taping is a relatively low-cost and simple procedure, reducing the use of drugs and showing encouraging prospects for the postoperative care after extraction of impacted mandibular third molars.

### Limitations

Our findings should be interpreted with caution due to the small sample size and the unequal number of men and women. Poor knowledge about kinesio taping limits the number of patients included in the study; however, this research is being continued on a larger scale with a larger sample size.

## 5. Conclusions

Kinesio taping in addition to NSAIDs was found to be more effective than NSAIDs alone in increasing the degree of jaw opening, decreasing pain intensity, and reducing the dosage of non-steroidal anti-inflammatory drugs in patients after impacted mandibular wisdom teeth surgery. The cooperation of dental surgeons and the physiotherapeutic team is associated with a measurable benefit, which is the reduction of the inflammatory process and a significant reduction in the use of non-steroidal anti-inflammatory drugs. It should be introduced as a standard in the surgery of impacted lower molars.

## Figures and Tables

**Table 1:** Calculation of Ganss ratio.

Ganss (A/B/C)	Control	Study	*p*-Value
A	3	4	0.89
B	6	6	
C	6	5	

**Table 2:** Characteristics of patients in the test (n = 15) and control groups (n = 15).

	Control Group	Study Group	*p*-Value
Age, years, mean ± std	29.73 ± 13.66	22.93 ± 5.02	0.235
Gender, female, n (%)	12 (80%)	9 (60%)	1.0
male	3 (20%)	6 (40%)	

**Table 3:** Comparison of the maximal mouth opening in the test group (n = 15) and the control group (n = 15).

	Study Group	Control Group	*p*-Value
MMO before surgery	46.86 ± 5.64	50.50 ± 6.57	0.128
MMO percentage change after 2 days	−0.40 ± 0.19	−0.52 ± 0.13	0.073
MMO percentage change after 7 days	−0.02 ± 0.05	−0.10 ± 0.09	0.0101

MMO—maximal mouth opening. VAS—visual analog scale comparison of pain perceived by the patients.

**Table 4:** Comparison of pain intensity according to the VAS—visual analog scale in the test group (n = 15) and the control group (n = 15).

	Study Group	Control Group	*p*-Value
VAS 1 day after surgery	5.64 ± 2.37	7.71 ± 2.16	0.0114
VAS after 2 days	3.07 ± 1.64	3.64 ± 1.82	0.391
VAS after 7 days	0.43 ± 0.65	0.71 ± 0.91	0.405

Comparison of the facial tissue swelling.

**Table 5:** Comparison of the facial tissue swelling in the test group (n = 15) and the control group (n = 15).

Sum	Study Group Mean ± sd	Control Group Mean ± sd	*p*-Value
Sum before surgery	466.57 ± 23.69	463.64 ± 27.50	0.765
Sum percentage change after 2 days	0.08 ± 0.03	0.07 ± 0.04	0.227
Sum percentage change after 2 days	0.02 ± 0.02	0.01 ± 0.01	0.963

Comparison of the use of NSAIDs. NSAIDs—non-steroidal anti-inflammatory drugs

**Table 6:** Comparison of the use of NSAIDs (100 mg ketoprofenum) in the test group (n = 15) and the control group (n = 15).

Med (Number of Tablets/100 mg Ketoprofenum)	Study Group Mean ± sd	Control Group Mean ± sd	*p*-Value
Med 1 day after surgery	0.86 ± 0.95	1.79 ± 0.80	0.0102
Med 2 days after surgery	0.50 ± 1.61	0.93 ± 1.00	0.0131
Med 7 days after surgery	0.07 ± 0.27	0.00 ± 0.00	0.317

Med—medications taken.

## Data Availability

The data that support the findings of this study are available from the corresponding author upon reasonable request. MDPI Research Data Policies.
